# Organoid biobanking, autonomy and the limits of consent

**DOI:** 10.1111/bioe.13047

**Published:** 2022-05-09

**Authors:** Jonathan Lewis, Søren Holm

**Affiliations:** ^1^ Department of Law, The Centre for Social Ethics and Policy University of Manchester Manchester United Kingdom of Britain and Northern Ireland; ^2^ Centre for Medical Ethics University of Oslo Oslo Norway

**Keywords:** authenticity, autonomy, biobank, competence, consent, liberty, organoid, precision medicine, regenerative medicine

## Abstract

In the debates regarding the ethics of human organoid biobanking, the locus of donor autonomy has been identified in processes of consent. The problem is that, by focusing on consent, biobanking processes preclude adequate engagement with donor autonomy because they are unable to adequately recognize or respond to factors that determine authentic choice. This is particularly problematic in biobanking contexts associated with organoid research or the clinical application of organoids because, given the probability of unforeseen and varying purposes for which a donor's organoids could be employed and given the different ways in which a donor can relate to her biospecimens, a donor can value her organoids differently in different contexts, and her reasons for autonomously permitting use of her cells and tissues in one case may not support an autonomous decision in another. In response, this paper has three aims: first, to make the case for why organoid biobanks ought to respect donor autonomy conceived as authentic choice; second, to explore the autonomy‐respecting limits of established and widely prevalent models of biobank consent; and third, to propose certain conditions that organoid biobanks ought to support or facilitate in order to respect donor autonomy.

## 1 INTRODUCTION

An organoid has traditionally been defined as a three‐dimensional structure that has been grown in vitro from stem cells, which self‐organize to take on the functional and structural properties of in vivo organs with organ‐specific cell types albeit without the defined general architecture typical of an organ.^1^ Whereas organ‐restricted adult stem cells (‘aSCs’) can be used to grow specific, predefined organoids, pluripotent stem cells, such as human embryonic stem cells (‘hESCs’) and induced pluripotent stem cells (‘iPSCs’) (obtained from skin or blood cells), can be used to develop any type of human organoid and thereby, in principle, imitate any type of tissue or organ in the human body.

So far, researchers have derived organoids of differing levels of maturity and complexity, conducted in vitro modelling of physiology, functionality and pathology for some organs/parts of organs, and developed animal models for toxicity and drug tests using organoids, organoid‐on‐a‐chip technologies or organoids in combination with other types of stem cells.^2^ In addition, patient‐derived organoids have been used to model pathologies of human genetic and congenital disorders and specific diseases, including Alzheimer's disease, frontotemporal dementia, cystic fibrosis, the Zika virus infection, and liver disease.^3^ Many of those undertaking organoid research anticipate that it will lead to clinical translation, particularly in contexts such as precision medicine. Given that organoids are anticipated to have the same functional and structural properties as the organs that they aim to imitate, one of the goals of organoid research is to determine whether they respond the same way to drugs as in vivo organs. If the response to a therapeutic agent can be replicated in an organoid, then there is scope to develop the organoid as a standard for modelling specific diseases, testing certain therapies, understanding human drug metabolism, and developing standardized, pharmacy‐ready drugs.^4^ In addition, there is the possibility of using organoids derived from individual patients in order to produce more personalized models of pathogenesis and to facilitate more personalized treatment regimens. However, although precision, personalized and regenerative medicine applications are foreseen in the future,^5^ the majority of the research on which such applications stand to be based is, at the time of writing, still in its preclinical phase, and there is not enough evidence to determine the feasibility of these applications in clinical medicine, their efficacy, risks, uncertainties and burdens, the suitability of current clinical trial protocols and drug development pathways for organoid technologies, or the universal standards for their design, fabrication and utility.^6^

Organoids and the cell lines from which they are derived can be stored in biobanks—collections of residual or research‐specific human biomaterials assembled for medical scientific research purposes. Indeed, organoids are ultimately dependent on biobanks for their distribution to multiple researchers and commercial partners across the globe.^7^ When it comes to questions of autonomy, the focus has predominantly been on the relationship between biobanks and those participants and patients that have donated or are considering donating their cells or tissue. Specifically, given the ethical and legal requirement for biomedical research involving humans to obtain informed consent, it has been the question of the form that biobanking consent processes *should* take that—at least when it comes to donor/patient autonomy—has attracted the most attention, both in the scholarly literature and in the realm of public policy and regulatory guidance. The problem is that, by focusing on consent, biobanking processes preclude adequate engagement with donor autonomy because they are unable to adequately recognize or respond to factors that determine authentic choice. In the next section, we will explain and justify a principled distinction between the concept of consent and the concept of autonomy with a view to, in the subsequent section, presenting a case for why biobanks and organoid biobanks in particular ought to respect donor autonomy conceived as authentic choice.

## 2 DISTINGUISHING CONSENT AND AUTONOMY IN BIOBANKING

In biomedical research and clinical contexts, informed consent performs four principal functions. First, it is the standard mechanism through which a patient exercises her liberty at law and consents to bodily interference.^8^ Second, whereas the consent dimension of informed consent protects patients from nonconsensual trespasses upon the body and is supported by the tort of battery,^9^ reasons for the disclosure of material risks and benefits associated with a specific research or clinical intervention have traditionally been dealt with by the law of negligence, and, to that extent, informed consent functions to protect biobanks, researchers and institutions from liability for injury.^10^ Third, when it comes to a donor's participation in biobanking processes, consent is the mechanism through which she articulates the boundaries for what she considers to be permissible use of her bodily material.^11^ Fourth, to the extent that a biobank or research studies related to the biobank involve the processing of personal information, explicit consent is one of the legal means through which they can permissibly use a participant's data in specific ways and generate new personal data through analysis of the donated material or derivatives of that material. (One might contend that consent is also a mechanism for the waiving of property or related rights, but this is not the norm in biomedical research contexts.)

Various models of consent have been proposed that individually give different degrees of weight to these specific functions. Some models, for example broad consent and consent for governance, prioritize donor liberty and the protection of bodily integrity. However, according to critics, they delineate types of material risk and benefit disclosure that fall below the appropriate standard for genuinely *informed* consent and limit downstream opportunities for donors to control which research projects and clinical applications can permissibly use their tissues, cells and associated organoids.^12^ Other models, for instance (project‐)specific consent, provide donors with greater choice in specifying cases of permissible use. However, given that (project‐)specific consent requires consent to be obtained for already collected biospecimens whenever a new study is proposed, critics argue that it risks impeding the utility of biobank research, for example by creating delays, by diverting research resources to consent acquisition, and through the increased likelihood of donor unresponsiveness.^13^

Given the amount of scholarly and regulatory attention that has been accorded the concept of consent, some critics have argued that too much normative weight has been attributed to the concept of autonomy, disproportionately influencing biobank structures, governance and processes. Some commentators have suggested that veracity (‘telling the truth’) should be prioritized by biobanks over and above autonomy, and, on that basis, an open or blanket consent model should be adopted that only allows donors to consent to the unrestricted use and disclosure of their health and genetic information, with no promises of anonymity or confidentiality.^14^ By contrast, Prainsack and Buyx have argued that because consent is primarily employed to manage participant risk, and the risks associated with current biobank‐based research are oftentimes small (relative to those encountered in other situations of disease research or clinical trials), participants should be afforded the opportunity, after the initial consent procedure, to voluntarily limit their autonomy and willingly assume the small levels of risk and uncertainty that this demands.^15^ It is claimed that such an approach allows biobanks to pursue a *solidarity* approach to governance that better assists the potential beneficiaries of biobank‐based research. It has also been claimed that respect for autonomy is fully compatible with a participant's voluntary consent to accept the (relatively small) costs associated with downstream limitations on her autonomy that inevitably arise through solidarity‐oriented biobank governance models.^16^

The problem with these sorts of criticisms of autonomy‐oriented biobanking processes is that they (implicitly) identify the locus of autonomy considerations in processes of participant consent. Moral psychology has shown that although consent *facilitates* participant autonomy, the typical conditions for informed consent cannot be equated with the conditions for the exercise of autonomy.^17^ Relatedly, obligations associated with respect for consent do not adequately account for the obligations derived from the principle of respect for autonomy. For instance, as Manson argues, the ‘informed consent paradigm is not about giving the participant the opportunity to shape the scope of her permission, or introduce terms and conditions on her permission’; rather, it is a ‘binary decision, a decision whether to consent or not’, which, if applicable, includes a right to withdraw.^18^ Accordingly, ‘this kind of “fixed” recruitment does not infringe upon, or disrespect the participant's liberty’.^19^ This claim is correct because what we are dealing with is the participant's *liberty* and not necessarily her *autonomy*.

Respect for autonomy is more normatively demanding, and this is because there are values to autonomy that extend beyond the values of informed consent, where the latter are typically understood as protections from moral and legal wrongs of (nonconsensually) violating a research participant's or patient's liberty at law, bodily integrity, sovereignty and permission.^20^ For instance, although Feinberg observes that the concept of autonomy can refer to a set of rights expressive of one's sovereignty over oneself, which closely resembles at least one of the primary functions of informed consent, autonomy has several other meanings in moral and political philosophy, including 1) the capacity to govern oneself, 2) the actual exercise and achievement of self‐government and 3) a personal ideal.^21^ For Feinberg, these differences in meaning extend to several different values, which, he notes, can include the connection of autonomy to moral and legal responsibility, autonomy as a necessary condition for equal political standing, and, most importantly where medical decision‐making and research participation is concerned, autonomy as a barrier to paternalism.^22^ In other words, autonomy is the ‘value that paternalism fails to respect’ because the latter involves ‘a judgment that the person is not able to decide for herself how best to pursue her own good’.^23^ We should also note that specific interferences that affect a patient's capacity to govern herself need not always be paternalistic; they can be malign (e.g., manipulative, oppressive, abusive, and so on) or just dismissive of a patient's choices or reasons.

To better understand the conceptual distinctions between consent and autonomy, we need to understand that philosophical accounts treat the concept of autonomy as constituted by two broad categories: competency and authenticity.^24^ ‘Competency’, ‘mental capacity’ and the ‘capacity for autonomy’ are often treated as having the same meanings such that, on the basis of a certain amount of consensus amongst medical ethicists, a competent or capacitous individual is considered to have the ‘capacity for reason’, that is, ‘the capacities to comprehend information, critically reflect on and revise beliefs, and make a decision in the light of information’.^25^ When combined with liberal principles, the concept of the competent agent has been the backbone of regulatory and statutory approaches to medical decision‐making.^26^ Specifically, a competent or capacitous patient is deemed at law to have fulfilled the necessary first‐person conditions for informed consent. However, the fact that an individual has been accorded the liberty to consent on the basis of her (presumed) competency/mental capacity does not guarantee that the consent she provides will be autonomous, because there are no assurances that she has, as a matter of fact, understood or rationally deliberated on the information that she has been given.^27^ Thus, satisfactory fulfilment of the conditions for competency (i.e., the capacity for reason) combined with the provision of—what a clinician or biobank deems to be—relevant information is insufficient to show that autonomy has been exercised in any specific decision.

This brings us to the second family of conditions for autonomy: authenticity. According to Christman, authenticity conditions are employed in accounts of autonomy in order to highlight the fact that genuine exercises of autonomy depend on the degree with which one's decisions, choices and actions are governed by—depending on the theory of autonomy to which one subscribes—reflection on, endorsement of, identification with or response to one's own values, desires, reasons or reasoning.^28^ As Coggon and Miola observe, when we are discussing whether an agent is exercising their autonomy, ‘there is a concern not just for the capacity for reason, but also for the effective use of it’.^29^ There are two key ways in which we can interpret the term ‘effective’ here. First, the effective use of an individual's capacity for reason can be understood in terms of ‘the soundness of her reasoning, given her own values’.^30^ Second, an individual can be said to effectively exercise her capacity for autonomy to the extent that she is the ‘power’ behind whatever reasoning directly gives rise to her decisions, choices or actions.^31^ Although there are philosophical disagreements about what exactly constitutes ‘sound reasoning’ and the nature of the ‘power’ over such reasoning, the point is that an agent's values, desires and reasons ‘can be more or less autonomous depending on whether the processes or volitional structures by which they come to be developed are *truly* her own’.^32^

Respect for consent demands recognition of an individual's permission or refusal if she is informed and satisfies certain legal competency conditions. However, the presence of authenticity conditions in theories of autonomy suggests that respect for the exercise of an individual's autonomy demands not only recognition of her status as a competent deliberator and as someone who has the ‘power’ to make normatively significant judgements about matters that concern her,^33^ but also recognition that she regards herself as having ‘legitimate reasons’ to raise claims to autonomy in relation to those matters.^34^ In other words, she recognizes that her decisions, choices or actions are warranted or deserved because she holds her own values, desires or reasons and considers them to be worthy of deliberation or a response. As Christman observes, because an individual's authentic decisions and choices are dialogically related to the respect she has for herself, this respect should be reciprocated by those to whom her decisions and choices are explicitly or even implicitly addressed.^35^

The key point where participation in biobanking processes is concerned is that autonomy ‘has value simply because it constitutes, in part, the human agency and capacity for *authentic* choice that grounds respect for ourselves and other persons’ [italics added].^36^ By contrast, focusing exclusively on the conditions required for consent leads to biobanking processes that preclude adequate engagement with the very question of the autonomy (or lack thereof) of donors. Accordingly, one of the reasons for disambiguating between autonomy and consent is that the former is concerned with the requirement to permit capacitous individuals to make authentic choices, that is, to effect changes in their lives in a manner that is consistent with the values, motivations and reasons that they themselves would voluntarily endorse, identify with or respond to.

## 3 THE VALUE OF AUTONOMY IN BIOBANKING AND ORGANOID RESEARCH

Even though a valid distinction can be drawn between the conditions that need to be met for an individual to deliver legitimate consent and the conditions for the exercise and achievement of their autonomy, one might still question whether the concept of autonomy should perform a substantive role in the ethical decision‐making and governance of organoid biobanks once a participant has consented to the donation of her tissue. In other words, why should biobanks in general, and organoid biobanks in particular, respect donor autonomy conceived as authentic choice (and not just rely on a thinner conception of valid consent)?^37^ In what follows, we offer three arguments to support the claim that biobanks ought to respect donor autonomy in this more normatively demanding sense, and we explain the limits and limitations of established models of biobank consent in terms of their respective abilities to meet these requirements. Two of these arguments can be applied to biobanks in general, and the third responds to some of the challenges posed specifically by organoid research.

First, biobanks are required to respect donor autonomy conceived as authentic choice if they are genuinely concerned with respecting donor autonomy. Contrary to this claim, some commentators have argued that because the risks to donors are so low in biobank research and the potential of a valuable outcome is so high then this a good reason to lower the usual standards for informed consent.^38^ However, as Hofmann, Solbakk and Holm demonstrate, there appears to be no valid argument in favour of this sort of ‘biobank exceptionalism’.^39^

One way to deal with the problem of biobank exceptionalism is for a biobank to adopt a broad consent approach, which has been perceived to operate on a scale ranging from consent to certain types or classes of research and clinical applications within initial broadly defined boundaries to blanket (i.e., unrestricted) consent to any kind of use, be it research, clinical or commercial.^40^ Depending on the terms of the initial consent, biobanks, researchers and clinicians can use a donor's biospecimens and data without obtaining new consent. However, one of the issues facing models of broad consent seems to be that individuals' consent to participate in biobanks cannot be fully informed because the very nature of biobanks is to collect samples for future research uses that may not yet be formulated and, most importantly, the risks of which are not known. The risks to a donor and the nature of those risks are morally relevant, but assessment of those risks requires specific knowledge about the ways in which biospecimens will be used in research. If an understanding of what the research involves and entails is necessary for consent in terms of determining risk, ‘then consent to biobank research of a general and unspecified kind cannot be obtained, neither of a narrow brand nor of a broad one’.^41^

Faced with the practical impossibility of obtaining genuine informed consent in biobanking contexts and the epistemic limits concerning future organoid research uses, it seems that biobanks have no choice but to look to alternative models of ethical engagement with their donors, such as consent for governance or models of broad consent that lean heavily on governance, the terms and values of which are stated at the time of seeking initial consent. According to these models, rather than, or, in the case of broad governance‐based models, in addition to consenting to a range of research uses and clinical applications, donors are asked to consent to a specified governance infrastructure and associated governance obligations that will decide on the use of the donated material if and when new questions arise in the future.^42^ Accordingly, future developments in organoid‐based technology, research and therapy are dealt with by a combination of ethical oversight and participant engagement, with the latter, for instance, consisting of groups of donors or the wider public engaging directly, deliberatively or representatively in the design and continuous adaptation of biobank governance.^43^ Not only do participant engagement and ethical oversight function as safeguards to ensure that the interests of different stakeholders are taken into account, they can contribute to decisions about which biobank activities require new consent.^44^ However, consent for governance cannot achieve a goal of protecting the autonomy interests of individual biobank donors.

First, as advocates of the consent for governance model acknowledge, these proposed governance mechanisms preclude adequate engagement with individual autonomy because participant engagement and ethical oversight have a collective focus, meaning that the interests that are respected in biobank decision‐making are *collective* interests and not necessarily those of individual donors.^45^ Second, alternative consent frameworks that prioritize collective interests rather than individual interests in the name of group majority can be conceived of as hard paternalism if the collective interests are truly shared by all.^46^ If the collective interests are not fully shared, this is not even paternalism, but pure majoritarian use of power. Third, given that the success of governance‐based consent frameworks is, in part, determined by donor trust in the biobank and its governance,^47^ the ability of a biobank to achieve its aims does not preclude recognition of and respect for a more normatively demanding conception of autonomy than is commonly assumed by models of consent.^48^ Indeed, as we will argue in relation to the second argument for why biobanks ought to respect authentic choice, developing trust between biobanks and would‐be donors does, in certain circumstances, require the former to consider and respect individual values. Therefore, although the facilitation of good biobank‐based research governance is not, in itself, necessarily incompatible with respect for autonomy, that does not mean that the autonomy‐respecting dimensions of biobank processes can be framed solely in terms of consent for governance. Given the autonomy‐respecting limits of consent‐based models, respect for donor autonomy or, pace O'Neill,^49^ promotion of donor trust in biobanks necessitates a move away from an approach to autonomy based on limited downstream control and the transfer of information that the biobank deems to be relevant towards an approach based on the donors' individual values and motivating attitudes.

The second argument for why biobanks ought to respect donor autonomy conceived as authentic choice turns on evidence that suggests that such an approach may be required in certain instances for biobank research to achieve some of its aims. Recognizing the problems with mandatory (project‐)specific consent and the applicability of the notion of *informed* consent in biobank contexts, advocates of consent for governance or broad, governance‐based consent frameworks have observed that a primary aim of a biobank is to encourage practices of solidarity on the part of biobank researchers, members of a biobank's governance team, and its donors in order to support, facilitate and undertake research that assists and benefits others.^50^ It is reasonable to suggest that the success of a biobank in meeting this aim relies in no small part on the willingness of participants to donate time, bodily materials, data and information. To that end, advocates of governance‐based consent models accept that such models depend upon a degree of donor autonomy to the extent that donors actually value the ‘value system’ of the biobank, and, should the value system or the goals of the biobank change significantly, are afforded the opportunity to opt out. In addition, such models rely upon autonomous decision‐making to the extent that donors perceive these values as trumping others, such as more control over the future use of their biospecimens or being informed individually about incidental findings that may be relevant but not medically actionable. On the basis of these aims and autonomy considerations that are often acknowledged by advocates of consent for governance or broad consent, there is scope to suggest that would‐be donors who do not value the value system of the biobank, or who have specific interests and values that they believe are not being recognized or respected by a particular governance model, will be unlikely to participate. And attempts to coerce individuals into donating to a biobank whose goals and value system they do not value would go against the governance principles of any morally reasonable biobank.

Studies have provided evidence that suggests that there are challenges facing biobanks in terms of achieving their primary aim, and, in seeking to overcome some of these challenges, particularly those that turn on the participation of individuals from minority ethnic, social and cultural groups, that biobanks ought to engage with and, ultimately, respect their values. Highlighting this issue, evidence suggests that there is an association between minority status, mistrust and reluctance to donate biomedical samples.^51^ Driving this reluctance are multifaceted considerations springing from individual moral values, cultural and religious values, and historical experiences of exploitation in general and specifically as research subjects.^52^ Furthermore, where such issues concern models of donor consent, studies that have engaged with members of minority groups have provided evidence suggesting that consent processes do not address participants' moral, religious and cultural concerns about the use of their biomaterial and that acknowledgement of, and respect for, individual participant values is an essential component of establishing trust between donors and biobanks.^53^ On that basis, participants from certain minority groups have stated that they do not perceive consent for governance or broad, governance‐based consent as substitutes for autonomous decision‐making, or vice versa.^54^ Ultimately, respect for autonomy and broad consent/consent for governance are taken to serve different functions, and the success of biobank governance (where the involvement of individuals from minority groups is concerned) depends on the biobank's capacity to respect those decisions that demarcate permissible use of an individual's biospecimens.

The question remains, however, why should biobanks respect the autonomy of members of minority groups when it is apparent that they would want to see biobanks acknowledge and respect their authentic choices? Let us accept the evidence that suggests that members of certain socio‐cultural groups (like everyone else) have individual moral values and religious and cultural values that inform their decisions about whether to donate biomaterial and to which research studies they would permit the use of their tissues and cells. If that is the case, then proponents of consent models that do not facilitate explicit engagement with these values on an ongoing basis or advocates of broad, governance‐based consent models that do not allow individual participants to shape the terms of their initial consent in downstream situations must be confident that the risk of their nonparticipation will not undermine the validity of research results or the applicability of research products owing to the limited diversity of the biospecimens deposited in a biobank. Given the diverse range of clinical applications of organoid research and technologies that are currently anticipated, particularly in the modelling and treatment of genetic diseases, and given that the majority of organoid research and application necessitates genetic sequencing, there are risks that a lack of diversity within biobank material could limit our understanding of certain pathologies, for which organoids are being employed to model, screen and develop more precise and more personalized therapeutics.^55^ In addition, a lack of diversity in the deposited biomaterial could lead to the generation of research‐based clinical products that would only be relevant to certain sections of the population, thereby exacerbating inequities and health inequalities.

The third argument for why organoid biobanks should approach and respect donor autonomy in terms of authentic choice is based on the claim that respect for a donor's liberty via a consent‐based approach is an insufficient measure for protecting would‐be donors from coercion or other forms of undue influence. As O'Neill argues, researchers and biobanks have obligations not to deceive, coerce or unduly influence donors, and advocates of informed consent suggest that one of its functions is to limit these sorts of influence.^56^ She also argues that if biobank researchers do not accept this obligation, then the alternative is a paternalistic approach to donor participation. As Hofmann, Solbakk and Holm observe, there are defendable versions of paternalism, but most of them are based on the principle of beneficence: an intervention in a person's life can be justified (without consent) because the benefits for this person outweigh the risks.^57^ The problem with employing paternalism as a means to enrol would‐be donors in the biobank or to ensure that donors contribute their biomaterial for some or all future research uses is, as we observed above, that organoid biobanks and researchers are unable to assess those risks and benefits owing to inadequate knowledge about the ways in which biospecimens and derived organoids will be used. As a result, a paternalism based on a trade‐off between benefits and risks seems not to be justifiable (or perhaps even possible).

For organoid research, the problem, where coercion and undue influence is concerned, is not solely that this is a ‘new field’ for which the desired research and clinical applications remain speculative and that, therefore, accurate benefit–risk profiles necessarily cannot be provided. The problem of coercion arises in organoid research contexts because, although the development of organoids requires the manipulation of donated cells or tissues and considerable expertise, effort and investment applied to those biospecimens, the relationship between a donor and organoids derived from their biospecimens can have moral value. As organoid researchers themselves have acknowledged, organoids relate to the bodily integrity of donors, not only in terms of genetics, but also in the sense that they represent the (dys)functioning of the bodies of their donors.^58^ On that basis, organoids are perceived to complicate the issue of what does and does not form part of a human body. Given the current stage of organoid derivation and research, some donors may relate to organoids derived from their biospecimens only as tissue samples or living cell lines.^59^ However, it has been argued that such relations are likely to become increasingly complicated and the boundary between organoid and body increasingly blurred when, as researchers anticipate, organoids become more mature and complex to the point that they develop into fully functioning mini‐organs or organ systems.^60^ It might sound strange to conceptualize something as part of the body that is clearly outside of the body proper and that has never been part of the body. However, it is, in reality, no more strange than the view posited by the idea of ‘extended cognition’ that we should conceptualize some of the electronic devices to which we have, for instance, outsourced memory tasks as part of our extended brain. Second, organoids relate to the personal identity of donors. For instance, organoid‐based research is likely to reveal the donor's genetic make‐up, given that sequencing techniques are routinely applied. Not only can this generate study‐specific information about a donor's present clinical conditions, which can then be used for diagnostic, prognostic and possible treatment purposes, it can also uncover findings unrelated to the study question, such as a donor's risk of hereditary disease derived from the presence of certain genetic contributors.^61^ Such findings can shape and reshape the meanings and attitudes that donors attribute to their disorders, and, for that reason, organoids can be perceived to form both a literal and a symbolic representation of donors and their bodies.^62^ Furthermore, as commentators have argued, given that the aforementioned genetic, functional and meaning‐based relations between donors and their organoids call into question the categorization of organoids as purely ‘objective’ material over which those that have donated cells and tissue should have no moral claims, organoids can not only be valued by their donors, but also function as a stand‐in for the values and beliefs of those that donate.^63^

If we accept that the relationship between donors and organoids can have moral value, then the multifaceted nature of organoid research raises ongoing concerns regarding biobank coercion or undue influence as separate projects utilize those same organoids. For example, if, on the basis of a broad, blanket or governance‐based approach to consent, the biobank considers donor consent to organoid derivation research to entail consent to certain types of precision or regenerative medicine research that may be foreseen or unforeseen at the time of obtaining initial consent, and if the donor does not know that the former entails the latter, then it cannot be assumed that she consents to it. In addition, researchers, in principle, may know that bench research that seeks to derive organoids may be causally related to, for example, the transplantation of organoids after long‐term maturation, vascularization, and so on. Indeed, research proposals and projects that undertake organoid‐derivation research often state that discoveries at the bench will entail other kinds of research, such as disease etiology modelling, drug testing, toxicity testing or organoid transplantation in animals, in the application of organoid technologies.^64^ However, donors may be ignorant of the causal link, and even though they consent to studies that seek to derive organoids from their cells or tissue, they may not consent to those uses that researchers expect to follow as a matter of practical necessity. The point is that when a donor consents to a proposition describing an intended research use of their biospecimens or their organoids, neither the proposition's logical implications nor the causal links between one research use and subsequent uses is transparent. Thus, as a matter of principle, a donor cannot consent to them. As O'Neill argues, the logic of propositional attitudes, of which consent is a type, is such that if the initial research use is taken to entail certain other uses, obtaining consent for those studies is necessary both from a logical perspective and to avoid donor coercion or undue influence.^65^

This issue is particularly pertinent in cases involving the participation of ‘patient‐donors’ in organoid biobanks. Because many researchers anticipate that organoids will be used for pathology modelling, therapeutic screening and the development of transplant technologies, donors can also be current or prospective patients, who might believe that their health and wellbeing stand to benefit from any resulting therapeutics tested on, or developed from, their donated cells and tissues, or from transplantation technologies involving organoid‐derived organs, organoid grafts or organoids‐on‐a‐chip. This is not to suggest that by agreeing to participate in organoid research by donating to a biobank, patient‐donors are agreeing to any resulting treatment. As a result, at least from a medical ethics perspective, there is no obligation for the biobank to provide patient‐donors with the information that would be required as part of the clinical treatment process. That said, the anticipated clinical applications of organoid research may not materialize, or they may materialize in the future but not in time to treat patients who have donated their biospecimens. This poses risks for patient‐donors. For instance, they may have certain expectations about benefiting directly from the clinical translation of research that utilizes their organoids, and these expectations might inform their decisions about whether to pursue alternative treatment options available to them in the meantime. If a patient‐donor is participating in an organoid biobank on the basis that they value the desired outcomes in terms of their own health and wellbeing, then, in order to avoid coercing or unduly influencing them into providing their biospecimens, organoid biobanks have an obligation to provide information commensurable with those values, which, broadly speaking, might include details regarding the preclinical state of current organoid research, the absence of evidence from clinical trials, and the challenges facing not only the development of organoid‐derived therapeutics, but also the derivation of organoids suitable for precision or regenerative medicine application.^66^ Or to put it slightly differently, just like many other types of clinical research, organoid research may give rise to a therapeutic misconception among potential participants, which will then raise the same issues in relation to the validity of consent that therapeutic misconceptions do in other research areas.

The problem, where the potential for biobank coercion and undue influence is concerned, is not just that donors may be ignorant of the causal links or entailing relationships between different organoid‐based research activities or information regarding the likelihood of them benefitting from organoid technologies—an issue that undermines their ability to provide genuine, up‐front consent. If we accept that organoids can have moral value, in part because they function as a stand‐in for the values and other attitudes of their donors, then there ought to be moral limitations on what biobanks and associated researchers can and cannot do with a donor's organoids. And those limits may be established in no small part by the attitudes of donors towards the ethical issues to which organoid research and the development of organoid‐based clinical applications give rise. The multifaceted nature of organoid research and the diversity of anticipated clinical applications highlight several ethical concerns that are, in many ways, specific to the field of organoid research. Given that pluripotent stem cells can be employed to derive any type of organoid and thereby, in principle, yield any of the clinical applications that are currently anticipated, there is the potential for the decisions of every organoid donor regarding permissible use of their biomaterial to be affected.

Research involving gastruloids, which are cultured from either hESCs or iPSCs and which recapitulate processes of embryo development, raises ethical concerns regarding the use and destruction of hESCs, the moral status of the developing complex entity, the extent to which such an entity is allowed to mature, and the limitations of regulations and policies relating to this kind of research.^67^ Relatedly, and although bench research is not at the point of developing cognitive and sensory functioning entities, there is a major push to overcome certain limitations (e.g., the problems of oxygen and nutrient diffusion, the absence of a peripheral nervous system, and the problems of modelling interactions between different parts of the brain and understanding the neural activity of brain organoids) in order to generate next‐generation human cerebral organoids with greater degrees of complexity and maturity.^68^ This has led to debates concerning the possible sentience of future mini‐brains, that is, their capacity for experiencing feelings such as pleasure and pain, and, as a result, the ethical permissibility of conducting research that may, intentionally or not, yield organoids with sensory and/or cognitive capacities.^69^ As noted by the International Society for Stem Cell Research (ISSCR) in its recently revised *Guidelines for stem cell research and clinical translation*, there has been extensive media coverage regarding research into cerebral organoids and the question of whether such entities could achieve sentience, consciousness and/or cognitive function. Although there is no evidence at this stage to support such concerns, the ISSCR claims that organoid biobanks and researchers should be cognizant about the ethical issues that donors and other stakeholders may have regarding research into brain and cerebral organoids, particularly as the latter become more complex through long‐term maturation.^70^

Where precision medicine research involving organoids is concerned, one of the aims is to link donors' biospecimens with health, genetic and digital data in order to develop models of pathogenesis and therapeutic screening that take into account individual variability. This could directly impact patients' personal care in terms of diagnosis, prognosis, and/or treatment.^71^ A donor may have legitimate concerns regarding the use of her biospecimens for the identification and analysis of *collateral* findings (i.e., those unrelated to the study question) that could not, at the time of initial consent, have reasonably been foreseen or methodologically planned for. Second, whether or not she chooses to donate her tissue or cells for the purposes of precision medicine research might depend on the type of disclosure policies offered by the organoid biobank, both for study‐specific and incidental findings. Participants could be motivated to donate on the basis that the disclosed findings might help guide prevention and treatment, benefit family members, allow them to make life plans, lifestyle changes and reproductive decisions, or simply because they noninstrumentally or recreationally value knowledge of their genetic status.^72^ However, as Ploug and Holm observe, the issue is that the current consensus among experts favours disclosing only medically actionable findings.^73^ Not only does such an approach raise normatively relevant questions in and of itself, but also, where participant autonomy is specifically concerned, decisions will be made on behalf of donors to determine what types of results are disclosed to them.^74^

Although the clinical transplantation of organoids is still in a preclinical phase,^75^ the strategy of transplanting organoids for repairing or even replacing damaged tissues and organs builds on scientific principles already governing stem‐cell‐based treatments, some of which have reached human clinical trials for stroke, traumatic brain injury, and Parkinson's disease.^76^ Where in‐human trials are concerned, organoid transplantation raises a number of ethical issues that could influence a donor's decisions regarding what constitutes permissible use of her tissues and cells. For one, the first clinical trials will proceed without prior in vivo data from humans and relatively little (pre‐)clinical knowledge, and may pose unexpected risks (e.g., uncontrolled and undirected growth).^77^ In addition, regenerative medicine employing organoid technology has, in principle, the capacity to influence moral actions, experiences, perceptions, and quality of life in unanticipated ways.^78^ Moreover, before in‐human trials could begin, the only other plausible situation in which organoids could be linked to in vivo organs is through animal modelling, leading to the creation of a ‘chimera’, namely an organism carrying cell populations derived from two or more (genetically distinct) sources. Empirical studies have already shown that some donors would disapprove of certain sensitive applications of organoids, including chimeric research.^79^ With increased public scrutiny of research involving chimeras,^80^ there are scientifically principled—not to mention aesthetic—reasons that could influence a potential donor's decision to deny the use of her biospecimens in animal transplantation research. Much discussion has focused on the question of whether animals transplanted with human brain organoids would become more ‘human’, specifically, whether the resulting ‘extreme chimeras’ would be self‐aware, conscious and/or higher‐order cognitively functioning.^81^ However, it is argued that such concerns are not supported by the current state of the science and are thereby less germane to the immediate issue of (brain) organoid transplantation.^82^ By contrast, potential donors may have legitimate ethical concerns regarding the welfare of chimeric animals, and these welfare concerns may extend to chimeric research in general or, by framing discussions of brain organoid transplantation in terms of ‘specific brain enhancements in chimeras’, to the uncertainty surrounding the effects of transplantation on neurological and neuropsychiatric processes, including whether these manifest as pain, distress, anxiety, and so on.^83^ Alternatively, a potential donor may find the idea of implanting a human brain organoid into a monkey to be just plain odd or weird.

Given the values that individual donors may possess in relation to the specific ethical issues associated with organoid research, and given the multifaceted nature of organoid research and the epistemic limits regarding the prediction of the future of organoid research and technology, donors, at the time of enrolling, might have a high degree of confidence in their reasons for permitting the use of their cells in order to derive certain organoids, but they may not have enough information to commit to certain forms of future precision or regenerative medicine research. What is of normative significance is not so much the giving of consent, but the fact that a donor will be deliberating on values and reasons that are particularly susceptible to causal influences and change. As research, technology and associated regulations and guidelines develop, new and contextually relevant information will inevitably become available, concerning not only the use and application of a donor's organoids, but also the ethical issues associated with such uses and applications. Thus, if it is the case that a donor values the ethical concerns that arise from different organoid research activities both at the time of enrolment and in the future, and if we accept that organoids can have moral value on the basis of their genetic, functional and values‐based links to those that donate, then, in order to avoid coercing or unduly influencing a donor into participating in particular organoid activities to which she objects on ethical grounds, there is the need for biobanks and researchers to provide her with value‐sensitive information so that she can make a genuine, voluntary choice. Nevertheless, an advocate of broad consent may contend that the problem can be overcome by merely providing a donor with details of known or anticipated ethical issues arising from certain organoid research activities and asking the donor to consent to those activities on the basis of the information that is currently available. This, however, does not address the problem that could arise if unforeseen ethical issues were to arise downstream of initial consent. Furthermore, if we accept that organoids can have moral value, then we need to acknowledge that donors may relate differently to their organoids at different times, whether that means valuing organoids (and/or their constitutive parts) differently at different stages of research and clinical application or in relation to specific research projects, or whether that means the changing relations between a donor and her organoids as her values and motivating attitudes change over time. Empirical studies have shown that research participants perceive a connection to their organoids and, more importantly, the strength and qualitative nature of that connection depend on the type of organoid derived from their tissue and cells (e.g., stronger connections would likely exist for brain and gonadal organoids).^84^ The point is that broad consent or consent‐for‐governance, in and of themselves, cannot achieve the levels of information transparency and downstream control needed to address participants' potentially changing values concerning the use of their biospecimens over time.^85^ Nevertheless, an advocate of broad consent may rightly point out that a donor does have the opportunity to withdraw her samples and/or revoke her permission. However, this is not an option to withdraw and/or revoke on a case‐by‐case basis: ‘if the framework is a broad consent one, then the right to revoke is a generic, broad one’.^86^ In other words, withdrawal is all or nothing.^87^ Of course, the choice to withdraw may be nothing more than a compromise for the donor, made because of a lack of adequate choices, which would otherwise have been available under another model of consent, rather than a decision that reflects her own motivating attitudes towards organoid research or the clinical application of such research in general. Furthermore, unless the biobank were to stipulate irrevocable consent, it has an interest not to act in a way which would lead to large‐scale withdrawals. This situation could be exacerbated in the future if laws and regulations were to recognize that donors have certain moral claims to the organoids derived from their biospecimens and thereby allowed donors to withdraw their organoids rather than just their cells or tissues. Regardless of whether such a situation materializes, the interest biobanks have in avoiding large‐scale withdrawals of biospecimens is a reason for permitting donors greater levels of control than was necessarily available when initial consent was obtained.

Even though the relationship between donors and organoids has moral value, and even though the multifaceted nature of organoid research and application calls into question the ability of biobank consent to avoid coercion and undue influence, it still may be unclear why organoid biobanks are obligated to respect donor autonomy qua authentic choice. The previous discussion illustrates at least two problems where the potential for coercion and undue influence within organoid biobanking is concerned. First, whether it is because a biobank adopts a consent‐based approach that precludes tailoring of information for specific donors or because of the epistemic limits regarding future organoid research, there can be insufficient information for the donor to make judgments regarding causally related or entailing types of organoid research and/or to specify types of biospecimen use. Second, broad and governance‐based consent models for biobank participation can undermine the donor's ability to shape the terms of her initial consent.

In terms of the first issue, the problem with a one‐size‐fits‐all approach to the provision of information via a particular model of consent is that, no matter how detailed that information may be, it is considered to be a form of paternalism precisely because, in principle, it precludes engagement with the question of what a would‐be donor would need in terms of information in order to make a voluntary choice, which is commonly taken to be a necessary condition of informed consent.^88^ By providing a donor with information that the biobank deems to be relevant, the voluntariness of a donor's decision to consent to the stipulated uses of her cells, tissue and organoids can be called into question. Therefore, if information is available regarding the ethical issues associated with those uses and the kinds of research that researchers believe are entailed or causally related to the research to which the donor has agreed, and if the donor values that information, then providing her with that information will ensure that she is not coerced. The same claim applies to those donors who do not value any of the ethical issues to which organoid research gives rise but whose reasons for donating or not depend on the provision of information regarding the anticipated benefits of organoid research for the wider community. But a critic may well contend that if what we are solely concerned with is providing donors with the information they value so that they can specify permissible use of their organoids in accordance with their motivating attitudes, then (project‐)specific consent frameworks already facilitate a donor's exercise of autonomy in this specific sense. But the issue is not that straightforward. Some donors will inevitably have different values, motivations and reasons for permitting or denying the use of their biospecimens for *specific* research projects and clinical applications, and, as a result, they will require specific, value‐sensitive information regarding the ways in which their organoids will be used. However, even if this calls into question a one‐size‐fits‐all approach to information provision, it is just one domain in which autonomy is valuable to the donor. The point is that donors might also have values and interests relating to the nature of the decision‐making processes themselves. Respect for autonomy conceived as authentic choice should not be merely equated with the provision of specific‐consent processes because, just as some participants may hold values towards specific research projects and clinical applications that make use of their organoids, others may be interested primarily in assisting the potential beneficiaries of biobank‐based research and, therefore, wish to receive only ‘general information about requirements on biomedical studies involving human beings’ with a view to participating in a broad or governance‐based approach to biobank decision‐making.^89^ As O'Neill argues, there is no principled reason to believe that a donor has been coerced or unduly influenced even if she decides on the basis of limited information, which she, nevertheless, has requested, and has judged that such information is sufficient to reach a decision.^90^ Furthermore, in terms of the ability of individual donors to differentiate the terms of consent if and when their attitudes towards their organoids or the research goals and biobank values change over time, O'Neill demonstrates that they are not coerced and know that they are not being coerced when they understand that they can, at any time, change their decisions about participating in particular kinds of research or clinical applications or change their information preferences.^91^ To that extent, and notwithstanding its feasibility and scientific justifiability (or lack thereof),^92^ anonymization or deidentification of a donor's sample (traditionally discussed in the context of the ‘consent or anonymize’ paradigm)^93^ precludes any downstream recognition of the interests she may have in her tissues or cells that include and extend beyond an interest in privacy.^94^

On the basis of the preceding discussions, organoid biobanks ought to give donors control over the amount and content of information that they receive and provide them with ongoing opportunities to change their decisions in order to avoid coercion and undue influence. As we will demonstrate in Section 4, by meeting these obligations, biobanks are fulfilling two of the necessary conditions for respect for donor autonomy conceived as authentic choice.

## 4 RELATIONAL FACTORS BEHIND RESPECT FOR DONOR AUTONOMY IN ORGANOID BIOBANKING

As the preceding section demonstrates, there are reasons to suggest that donors' decisions regarding permissible use of their biomaterial are susceptible to causal influences. Specifically, when it comes to organoid biobanking, the theoretical basis of this awareness translates into an obligation for biobanks to provide information relating to the proposed use of a donor's organoids that she herself values.

Theorists of personal autonomy have argued that interpersonal relationships, which extend to the ways in which individuals are treated through communicative acts such as information transfer, are part of the ‘background requirements’ for the exercise and achievement of autonomy.^95^ Providing value‐insensitive information can causally contribute to the *authenticity* of a donor's decisions to donate her samples and for which purposes, to the extent that the values, desires and motives that she ends up endorsing or responding to are her own.^96^ It has been argued that the kind of paternalism expressed by the employment of a one‐size‐fits‐all approach to the provision of (medical) information compromises an individual's power over the reasoning processes that govern her decisions and choices, such that respecting decisions made in light of the presentation of information that a donor does not value would not be consistent with respecting her autonomy.^97^ Furthermore, according to certain accounts of the nature of autonomy, the exercise of an individual's autonomy demands self‐recognition of the fact that she has legitimate reasons to raise claims to autonomy in relation to, for instance, permissible use of her organoids. Accordingly, presenting a donor with biobank‐based research information that she does not value can undermine her autonomy because it suggests that her values are invalid or unworthy of consideration in decision‐making situations. This can compromise a donor's trust or confidence in, or commitment to, the reasons for her decisions, it can undermine her ability to respond to those reasons that she sees as valuable to pursuing some ends and not others, and it can affect the value she attributes to those decisions for the purposes of exercising her autonomy.^98^ The point is that a lack of confidence or trust in, or commitment to, her reasons can affect the legitimacy of her power over her reasoning.

Given the fundamentally relational nature of a donor's motivations and her attitudes towards those motivations,^99^ a second key element of respect for autonomy in organoid biobanking concerns the facilitation of decision‐making processes that support a *diachronic* and *longitudinal* approach to the exercise of donor autonomy. One argument for this recommendation turns on the claim that the personal history of an individual is a contributing factor in the exercise of her autonomy.^100^ Specifically, authentic choices and decisions are deemed to be constituted, in part, by ‘retrospective validation’, that is, the evaluation of past motivating attitudes that gave rise to certain decisions or behaviours and validating those attitudes after the fact.^101^ According to Christman, personal history is captured in exercises of autonomy by an individual attending to the manner in which a specific value, desire or motivation has developed. He establishes a diachronic test of authenticity according to which an individual is deemed to be autonomous relative to some value, desire or motivation ‘if, were piecemeal reflection in light of the history of the factor's development to take place, she would not feel deeply *alienated* from the characteristic in question’.^102^ For Christman, to be alienated from a value or motive is to experience a negative affect relative to it, whether that may be complete repudiation of the motivating attitude, to experience a conflicted motivation or to feel constricted by the value in question.^103^ For example, let us assume that a donor, who is concerned with animal welfare in research, discovered that the only reason she agreed to permit the use of her organoids for all types of regenerative medicine research was because the biobank informed her that any such research would protect the welfare of those animals. The donor's autonomy would clearly be in question if she saw that the biobank had not provided her with information that was already widely available in the media regarding the possible effects of specific transplant experiments on the neurological and neuropsychiatric processes of chimeric animals, and this led her to feel conflicted about the reason for permitting the use of her organoids. We can see, therefore, that the test for authenticity on Christman's account is not only premised on a conception of the autonomous agent as diachronically structured; it also supports our previous analysis of autonomy‐based reasons for the provision of value‐sensitive information.

The previous section argued that, in order to avoid coercion and undue influence, biobanks should afford donors the opportunity to shape the terms of their consent in downstream situations and, accordingly, change their decisions about participating in particular kinds of research or clinical applications that make use of their organoids. From a theoretical perspective, such an obligation rests on a *diachronic* and *longitudinal* approach to the exercise of donor autonomy. As we suggested in Section 2, one way to understand the *effective* use of an individual's capacity for reason in exercises of autonomy is in terms of the soundness of her reasoning given her own values. When we are discussing soundness of reasoning in the context of accounts of autonomy that focus on the evaluation of motivating attitudes, what we are primarily concerned with is an individual's ability to ‘discern what “follows from” one's beliefs and desires, and to act accordingly’.^104^ Thus, an individual can be considered to have legitimate reasons for her decisions and choices to the extent that her values, motivations and desires are supported by the exercise of her capacity for reason. Of course, what is implied by such an approach to autonomy is the possibility that an individual's values and motives can be altered in light of her evaluations and, therefore, that she can autonomously change her mind.^105^ Accordingly, for biobanking decision‐making processes to respect donor autonomy, they need to take a longitudinal approach and facilitate opportunities to respond to a donor's change of mind if and when she discovers she has good reason to do so. Such reasons, according to certain relational theorists of autonomy, can be constituted as much by emotions, desires, imagination and embodied experience as by rational reflection.^106^ Furthermore, as we have observed, to respect the autonomy of all donors, and not just those who value decision‐making processes on a case‐by‐case basis, such opportunities should extend not only to donors' decisions regarding permissible use of their organoids, but also to decisions regarding the structure of the decision‐making processes themselves (i.e., decisions to change from case‐specific decision‐making to broad decision‐making, or vice versa, and so on).

The third key element of respect for autonomy in organoid banking concerns the notion of choice, specifically (1) the choice of what biobank, research or clinical information the donor receives so that she can authentically choose the terms, conditions, cases and situations for the permissible use of her organoids; (2) the choice regarding types of decision‐making processes (e.g., case‐specific, broad, blanket, and so on); and (3) the opportunity to choose differently with regards to (1) and (2), should the donor have reason to do so.

When it comes to autonomy, Raz has argued that choice is normatively significant in and of itself because ‘adequate range of options’ is one of the three components of autonomy (along with competency and authenticity).^107^ However, even if we do not consider adequate choice to be a distinct component of the nature of autonomy, our analysis of the autonomy‐based reasons for the provision of value‐sensitive information and the facilitation of a diachronic and longitudinal approach to biobank‐based decision‐making shows that the degree of choice that one has with regard to one's decisions can affect the authenticity of those decisions. Recall, first, that a one‐size‐fits‐all, value‐insensitive approach to information provision is considered to be a form of paternalism to the extent that it undermines donor autonomy by constraining the reasons that a donor is able to respond to.^108^ Second, one of the problems with broad consent and consent for governance is that they do not allow a donor to shape the terms of her consent in downstream situations. Should a donor disagree with a certain use of her organoids or the value‐system or goals of the biobank, the only option available to her is to withdraw, which, given the lack of choice, can result in a decision that does not adequately reflect her own motivating attitudes towards organoid research in general. Therefore, respect for autonomy in organoid biobanking contexts requires, first, supporting the conditions for donors to attend to, reflect on and respond to the values and motives that they take to be potentially legitimate considerations for their decisions regarding permissible use of their organoids. As we have seen, this requires that a donor be able to choose the information that she receives from biobanks. Second, rather than being presented with just the option to consent (broadly) or withdraw, a donor should, within reason, be provided with the opportunity to decide the scope, frequency and context of her decision‐making participation in general and, if necessary, to alter these in the future in accordance with her changing values and motives—a model akin to what Ploug and Holm call ‘meta consent’.^109^ The point here, as Raz observes, is that *adequacy* of choice relates to the variety, and not the number of options available.^110^

Previously, we argued that models of consent developed for biobanking purposes preclude adequate engagement with donor autonomy because they are primarily concerned with facilitating informed consent, the conditions for which cannot be equated with the conditions for the exercise of autonomy. There is, however, one notable exception—*dynamic consent*. This refers to a personalized, online or IT‐based communication and interactive decision‐making interface that facilitates not only donor decision‐making, but also communication and information transfer between biobanks, researchers and donors.^111^ The interface and the principles on which it is based were developed, in part, because focus groups found that some biobank participants wanted more information about, and stronger oversight concerning the uses of, their samples and data.^112^ At the same time, developers of dynamic consent acknowledged that donor decision‐making in these respects could be better supported by bolstering communication and engagement between biobanks and their donors.^113^

When it comes to adequate choice, dynamic consent, in principle, allows donors to ‘drift’ between consent models, and thereby inherit the benefits of each, depending on the context.^114^ In other words, participants can provide different types of consent for different types of studies and/or clinical applications.^115^ For instance, they could opt to make decisions on a case‐by‐case basis for regenerative medicine research, but adopt broad consent for precision medicine research and blanket consent for organoid derivation research. Alternatively, participants could opt for case‐specific, broad or blanket consent for all potential uses. In addition, one of the core benefits of dynamic consent is the emphasis it places on timing. Recognizing that biobank research can take place over many years, advocates have demonstrated that an online or IT‐based decision‐making interface provides biobank donors with opportunities to receive new and accurate information regarding the uses of their biomaterials on an ongoing basis, to be reminded of the projects to which they have contributed, to check in at their own convenience, and to reconsider their decisions at a time and in a place that best suits them.^116^ It follows that dynamic consent, in principle, supports a longitudinal and diachronic approach to donor autonomy.^117^ Indeed, the approach is ‘dynamic’ because it allows interactions and responses over time if and when a donor decides that she has reason to change her decisions regarding permissible use of her organoids and/or her preferences for certain types of decision‐making process (e.g., when new research activities are proposed or when previously unforeseen information comes to light).^118^ In terms of information provision, and although one cannot guarantee that dynamic consent provides value‐sensitive information because whether it does or does not will ultimately be determined by the decisions and actions of particular biobanks, advocates have claimed that a dynamic consent interface can, in principle, provide donors and participants with the choice to receive tailored information to suit their specific values, motivations and interests.^119^ Finally, there are reasons to suggest that dynamic consent can assist biobanks in dealing with some of those issues on the basis of which we argued that they ought to respect donor autonomy conceived as authentic choice. For instance, proponents have argued that the facilitation and maintenance of an ongoing connection with donors might assist biobanks with fostering trust, especially as dynamic consent aims to promote improved understanding of research uses and outputs and to develop a stronger sense of collaboration between donors and researchers.^120^ In turn, such a flexible, dynamic and personalized approach to decision‐making might help to address moral, cultural and religious values that, as evidence suggests, cannot be adequately recognized by established models of biobanking consent and that lead to mistrust and reluctance to donate biomedical samples among minority groups.^121^

Despite these benefits, advocates of dynamic consent recognize that even this decision‐making tool is not without its drawbacks. First, given that study‐specific decision‐making would be made available to donors (along or in combination with broader models), there is the risk that a large volume of requests for (project‐)specific decision‐making could lead to a diversion of resources away from research activities, thereby affecting the ability of a biobank to operate at full capacity.^122^ Second, and despite the ability of dynamic consent to respond to and respect the authentic choices of donors, critics have suggested that, in practice, a digitally administered decision‐making process cannot ensure that the autonomy of potential participants is not being undermined as a result of the undue influences of friends, family members and other individuals or groups who have an interest in a donor's decisions.^123^ There are also concerns about the challenges an online or IT‐based decision‐making interface will create, particularly if it were to undermine inclusivity by excluding those individuals and groups who are unwilling or unable to use the technology or who do not have reliable access to the interface.^124^

Whether and to what degree organoid biobanking processes respect donor autonomy depends on whether and to what degree they recognize, incorporate and facilitate the conditions for *authentic* choice. Dynamic consent is a potentially appropriate model in this regard because, despite its name, it does not solely focus on the conditions for informed consent. More importantly, it (implicitly) captures those key elements of donor autonomy discussed above and is thereby, in principle, able to account for and respond to those factors that have the potential to support or undermine the authenticity of donors' decisions in specific instances.

## 5 CONCLUSION

When it comes to questions of autonomy, the focus of scholarly and public policy attention has been on the types of consent that biobanks should offer to donors both before and after initial donation of their biospecimens. However, whereas the values and functions of consent are predominantly understood in relation to the concept of liberty, the values of autonomy are to be found in relation to the notion of *authentic* choice. This means that the conditions of autonomy extend beyond the conditions of competency, mental capacity and/or the capacity for reason that, in part, ground respect for an individual's liberty. We have provided three arguments that attempt to justify why organoid biobanks are obligated to facilitate those conditions that are conducive to *authentic* decision‐making.

Critics of our approach to respect for donor autonomy in organoid biobanking might claim that it falls prey to the standard objection against (project‐)specific consent; specifically, that it risks impeding the utility of biobank research. In response, we should remind ourselves that the approach to autonomy adopted here is not to be equated with (project‐)specific consent. Indeed, we argue that donors, as part of having their autonomy respected, should be given the opportunities to decide whether to participate on the basis of specific, blanket, broad or governance decision‐making or a combination thereof (depending on the type of organoid research being conducted) and, indeed, to change their minds on an ongoing basis. The same claim applies to the information they receive from a biobank. If a donor values information regarding the ethical issues raised by current and future organoid research, then they should be provided with that information. Similarly, if a donor is happy to receive minimal information unless it relates to the anticipated benefits of organoid research for the wider community, then such information should be provided. As we have argued, the point is that to avoid coercion or undue influence and to meet their scientific aims in certain circumstances, biobanks ought not to provide donors with information that they do not value or limit the opportunities for donors to reshape the terms of their initial decisions unless it is the case that donors have agreed to participate on the basis of those limitations. At least where the participation of individuals from minority groups is concerned, there is evidence to suggest that such an approach is likely to increase biobank research utility rather than impede it. There are also reasons to believe that respect for donor autonomy conceived as authentic choice is required to minimize the risk of large‐scale biobank withdrawals. The point is that there seem to be no principled reasons for believing that such an approach to donor autonomy will make donors increasingly rare or unresponsive. In principle, donors who would only wish to partake based on a (project‐)specific decision‐making model are able to do so. The same applies to those who prefer a broad or governance‐based approach. However, we acknowledge that whether there is a risk to impeding a biobank's ability to operate at full capacity will, in practice, depend on the ability of biobanks to develop, implement, and manage a donor interface, akin to dynamic consent, that facilitates such a multifaceted approach to donor decision‐making or to conduct donor interviews that account for individual requests for information provision and ongoing decision‐making involvement. In addition, it will depend on the number of donors that wish to grant permission to the use of their organoids on either a study‐by‐study or other regular basis and, accordingly, on the capacity of biobanks to manage those decisions.

From a philosophical bioethics perspective based on principled considerations, do the most prevalent and established consent processes in organoid biobanking respect autonomy, tout court? They do not. There are limits to the dimensions of autonomy that can be respected by the most widely employed approaches to consent in organoid biobanking. The upshot of the approach taken in this paper is that to avoid prioritizing collective donor interests at the expense of the interests of individual donors, to avoid coercion and undue influence, and to promote biobank uptake among certain underrepresented socio‐cultural groups, biobanks ought to respect donor autonomy conceived as authentic choice. As a minimum, for those organoid biobanks that claim to respect donor autonomy based on established models of consent, such claims require careful qualification. Otherwise, they not only set unreasonable expectations for would‐be donors, but also, as we have seen, undermine their autonomy.

## Footnotes

1 Lancaster, M. A., & Knoblich, J. A. (2014). Organogenesis in a dish: Modeling development and disease using organoid technologies. *Science*, *345*, 1247125.

2 Ishiguro, T., Ohata, H., Sato, A., Yamawaki, K., Enomoto, T., & Okamoto, K. (2017). Tumour‐derived spheroids: Relevance to cancer stem cells and clinical applications. *Cancer Science, 108*(3), 283–289; de Miguel, M. P., Prieto, I., Moratilla, A., Arias, J., & Aller, M. A. (2019). Mesenchymal stem cells for liver regeneration in liver failure: From experimental models to clinical trials. *Stem Cells International, 2019*, 3945672; Aasen, D. M., & Vergara, M. N. (2020). New drug discovery paradigms for retinal diseases: A focus on retinal organoids. *Journal of Ocular Pharmacology and Therapeutics, 36*(1), 18–24; Schneemann, S. A., Boers, S. N., van Delden, J., Nieuwenhuis, E., Fuchs, S. A., & Bredenoord, A. L. (2020). Ethical challenges for pediatric liver organoid transplantation. *Science Translational Medicine, 12*(552), eaau8471; Shrestha, J., Razavi Bazaz, S., Aboulkheyr Es, H., Yaghobian Azari, D., Thierry, B., Ebrahimi Warkiani, M., & Ghadiri, M. (2020). Lung‐on‐a‐chip: The future of respiratory disease models and pharmacological studies. *Critical Reviews in Biotechnology, 40*(2), 213–230; Abdollahi S. (2021). Extracellular vesicles from organoids and 3D culture systems. *Biotechnology and Bioengineering, 118*(3), 1029–1049; Samimi, H., Atlasi, R., Parichehreh‐Dizaji, S., Khazaei, S., Akhavan Rahnama, M., Seifirad, S., & Haghpanah, V. (2021). A systematic review on thyroid organoid models: Time‐trend and its achievements. *American Journal of Physiology. Endocrinology and Metabolism, 320*(3), E581–E590.

3 Ming, G. L., Tang, H., & Song, H. (2016). Advances in Zika virus research: Stem cell models, challenges, and opportunities. *Cell Stem Cell, 19*(6), 690–702; Saini, A. (2016). Cystic fibrosis patients benefit from mini guts. *Cell Stem Cell, 19*(4), 425–427; Xu, M., Lee, E. M., Wen, Z., Cheng, Y., Huang, W. K., Qian, X., … Tang, H. (2016). Identification of small‐molecule inhibitors of Zika virus infection and induced neural cell death via a drug repurposing screen. *Nature Medicine, 22*(10), 1101–1107; Bredenoord, A.L., Clevers, H., & Knoblich J. A. (2017). Human tissues in a dish: The research and ethical implications of organoid technology. *Science, 355*(6322), eaaf9414; Bartfeld, S., & Clevers, H. (2017). Stem cell‐derived organoids and their application for medical research and patient treatment. *Journal of Molecular Medicine, 95*, 729–738; de Miguel et al., op. cit. note 2; Bowles, K. R., Silva, M. C., Whitney, K., Bertucci, T., Berlind, J. E., Lai, J., … Temple, S. (2021). ELAVL4, splicing, and glutamatergic dysfunction precede neuron loss in MAPT mutation cerebral organoids. *Cell, S0092‐8674*(21), 00829‐1; Chen, X., Sun, G., Tian, E., Zhang, M., Davtyan, H., Beach, T. G., … Shi, Y. (2021). Modeling sporadic Alzheimer's disease in human brain organoids under serum exposure. *Advanced Science, 8*(18), e2101462.

4 Dekkers, J. F., Berkers, G., Kruisselbrink, E., Vonk, A., de Jonge, H. R., Janssens, H., … Beekman, J. M. (2016). Characterizing responses to CFTR‐modulating drugs using rectal organoids derived from subjects with cystic fibrosis. *Science Translational Medicine, 8*(344), 344ra84; Ming et al., op. cit. note 3; Xu et al., op. cit. note 3; Chen, H. I., Song, H., & Ming, G. L. (2019). Applications of human brain organoids to clinical problems. *Developmental Dynamics, 248*(1), 53–64; Berkers, G., van Mourik, P., Vonk, A. M., Kruisselbrink, E., Dekkers, J. F., de Winter‐de Groot, K. M., … van der Ent, C. K. (2019). Rectal organoids enable personalized treatment of cystic fibrosis. *Cell Reports*, *26*(7), 1701–1708.e3.

5 Bredenoord et al., op. cit. note 3; Bartfeld & Clevers, op. cit. note 3; Chen et al., op. cit. note 4.

6 See references in note 2.

7 Boers, S. N., van Delden, J. J., Clevers, H., & Bredenoord, A. L. (2016). Organoid biobanking: Identifying the ethics: Organoids revive old and raise new ethical challenges for basic research and therapeutic use. *EMBO Reports, 17*(7), 938–941; Bredenoord et al., op. cit. note 3.

8 Lewis, J. (2021). Safeguarding vulnerable autonomy? Situational vulnerability, the inherent jurisdiction, and insights from feminist philosophy. *Medical Law Review, 29*(2), 306–336.

9 Archard, D. (2008). Informed consent: Autonomy and self‐ownership. *Journal of Applied Philosophy*, *25*(1), 19–34, 20.

10 O'Neill, O. (2004). Accountability, trust and informed consent in medical practice and research. *Clinical Medicine, 4*(3), 269–276; Coggon, J., & Miola, J. (2011). Autonomy, liberty, and medical decision‐making. *Cambridge Law Journal, 70*(3), 523–547, 533–535.

11 Manson, N. C. (2019). The ethics of biobanking: Assessing the right to control problem for broad consent. *Bioethics, 33*(5), 540–549, 543.

12 O'Neill, op. cit. note 10; Hansson, M. G., Dillner, J., Bartram, C. R., Carlson, J. A., & Helgesson, G. (2006). Should donors be allowed to give broad consent to future biobank research? *The Lancet Oncology, 7*(3), 266–269; Hofmann B. (2009). Broadening consent – And diluting ethics? *Journal of Medical Ethics, 35*(2), 125–129; Karlsen, J. R., Solbakk, J. H., & Holm, S. (2011). Ethical endgames: Broad consent for narrow interests; Open consent for closed minds. *Cambridge Quarterly of Healthcare Ethics, 20*(4), 572–583; Sheehan, M. (2011). Can broad consent be informed consent? *Public Health Ethics, 4*(3), 226–235; Helgesson, G. (2012). In defense of broad consent. *Cambridge Quarterly of Healthcare Ethics, 21*(1), 40–50, 44; Solomon, S., & Mongoven, A. (2015). Extending the surrogacy analogy: Applying the advance directive model to biobanks. *Public Health Genomics, 18*(1), 1–10; Manson, op. cit. note 11.

13 Mikkelsen, R. B., Gjerris, M., Waldemar, G., & Sandøe, P. (2019). Broad consent for biobanks is best – Provided it is also deep. *BMC Medical Ethics, 20*(1), 71.

14 Lunshof, J. E., Chadwick, R., Vorhaus, D. B., & Church, G. M. (2008). From genetic privacy to open consent. *Nature Reviews Genetics, 9*(5), 406–411.

15 Prainsack, B., & Buyx, A. (2013). A solidarity‐based approach to the governance of research biobanks. *Medical Law Review, 21*(1), 71–91.

16 Lensink, M. A., Boers, S. N, Jongsmaa, K. R., Carter, S. E., van der Ent, C. K., & Bredenoord, A. L. (2021). Organoids for personalized treatment of cystic fibrosis: Professional perspectives on the ethics and governance of organoid biobanking. *Journal of Cystic Fibrosis, 20*, 443–451.

17 Dodds,S. (2000). Choice and control in feminist bioethics. In C. Mackenzie & N. Stoljar (Eds.), *Relational autonomy: Feminist perspectives on autonomy, agency and the social self* (pp. 213–235). Oxford University Press; Mackenzie, C., & Rogers, W. (2013). Autonomy, vulnerability and capacity: A philosophical appraisal of the Mental Capacity Act. *International Journal of Law in Context, 9*(1), 37–52; Lewis, J. (2021). Autonomy and the limits of cognitive enhancement. *Bioethics, 35*(1), 15–22; Lewis, op. cit. note 8.

18 Manson, op. cit. note 11, p. 546. Although, on this line of arguments there seems to be no in principle reason why the right to withdraw could not be waived as part of what is consented to.

19 Ibid.

20 Archard, op. cit. note 9; Beauchamp, T. L. (2011). Informed consent: Its history, meaning, and present challenges. *Cambridge Quarterly of Healthcare Ethics, 20*(4), 515–523. Coggon & Miola, op. cit. note 10; Coggon, J., (2016). Mental capacity law, autonomy, and best interests: An argument for conceptual and practical clarity in the Court of Protection. *Medical Law Review, 24*(3), 396–414; Lewis, op. cit. note 8.

21 Feinberg, J. (1989). Autonomy. In J. Christman (Ed.), *The inner citadel: Essays on individual autonomy* (pp. 27–53). Oxford University Press.

22 Feinberg, J. (1986). *Harm to self. The moral limits of the criminal law* (vol. 3). Oxford University Press.

23 Christman, J. (2004). Relational autonomy, liberal individualism and the social constitution of selves. *Philosophical Studies*, *117*(1/2), 143–164, 157.

24 Lewis, op. cit. note 17, p. 16.

25 Ibid.

26 Ibid: 17; Lewis, op. cit. note 8, pp. 309–310.

27 Lewis, op. cit. note 8, p. 310.

28 Christman, J. (2020). Autonomy in moral and political philosophy. In E. N. Zalta (Ed.), *The Stanford encyclopedia of philosophy*. Retrieved from https://plato.stanford.edu/archives/fall2020/entries/autonomy-moral/

29 Coggon & Miola, op. cit. note 10, p. 528.

30 Ibid: 531.

31 Buss, S., & Westlund, A. (2018). Personal autonomy. In E. N. Zalta (Ed.), *The Stanford encyclopedia of philosophy*. Retrieved from https://plato.stanford.edu/archives/spr2018/entries/personal-autonomy/

32 Lewis, op. cit. note 17, p. 18.

33 Mackenzie, C. (2015). Responding to the agency dilemma: Autonomy, adaptive preferences, and internalized oppression. In M. Oshana (Ed.), *Personal autonomy and social oppression* (pp. 48–67, 55). Routledge.

34 Anderson, J., & Honneth, A. (2005). Autonomy, vulnerability, recognition, and justice. In J. Christman & J. Anderson (Eds.), *Autonomy and the challenges to liberalism: New essays* (pp. 127–149, 132). Cambridge University Press.

35 Christman, op. cit. note 23, p. 153.

36 Ibid: 153.

37 We wish to thank one of the anonymous reviewers for raising this question, and the three arguments we have developed below directly respond to their excellent comments.

38 Hansson et al., op. cit. note 12; Helgesson, G., Dillner, J., Carlson, J., Bartram, C. R., & Hansson, M. G. (2007). Ethical framework for previously collected biobank samples. *Nature Biotechnology*, *25*(9), 973–976.

39 Hofmann, B., Solbakk, J. H., & Holm, S. (2009). Consent to biobank research: One size fits all? In J. Solbakk, S. Holm, & B. Hoffmann (Eds.), *The ethics of research biobanking* (pp. 3–23). Springer.

40 Hofmann, op. cit. note 12; Petrini, C. (2010). ‘Broad’ consent, exceptions to consent and the question of using biological samples for research purposes different from the initial collection purpose. *Social Science & Medicine*, *70*(2), 217–220; Helgesson, op. cit. note 12; Solberg, B., & Steinsbekk, K. (2015). Biobank consent models – Are we moving toward increased participant engagement in biobanking? *Journal of Biorepository Science for Applied Medicine*, *3*(1), 23–33.

41 Hofmann et al., op. cit. note 39, p. 13.

42 Boers, S. N., & Bredenoord, A. L. (2018). Consent for governance in the ethical use of organoids. *Nature Cell Biology*, *20*(6), 642–645.

43 Boers, S.N., van Delden, J.M., & Bredenoord, A.L. (2019). Organoids as hybrids: Ethical implications for the exchange of human tissues. *Journal of Medical Ethics*, *45*(2), 131–139.

44 O'Doherty, K. C., Burgess, M. M., Edwards, K., Gallagher, R. P., Hawkins, A. K., Kaye, J.,… Winickoff, D. E. (2011). From consent to institutions: Designing adaptive governance for genomic biobanks. *Social Science & Medicine*, *73*(3), 367–374.

45 O'Doherty et al., op. cit. note 44; Prainsack & Buyx, op. cit. note 15; Boers et al., op. cit. note 43.

46 Hofmann et al., op. cit. note 39, p. 14.

47 O'Doherty et al., op. cit. note 44.

48 O'Neill, op. cit. note 10.

49 Ibid.

50 Prainsack & Buyx, op. cit. note 15.

51 Bussey‐Jones, J., Garrett, J., Henderson, G., Moloney, M., Blumenthal, C., & Corbie‐Smith, G. (2010). The role of race and trust in tissue/blood donation for genetic research. *Genetics in Medicine, 12*(2), 116–121; Prictor, M., Teare, H., & Kaye, J. (2018). Equitable participation in biobanks: The risks and benefits of a ‘dynamic consent’ approach. *Frontiers in Public Health, 6*, 253.

52 Prictor et al., op cit. note 51.

53 De Vries, R. G., Tomlinson, T., Kim, H. M., Krenz, C., Haggerty, D., Ryan, K. A., & Kim, S. Y. (2016). Understanding the public's reservations about broad consent and study‐by‐study consent for donations to a biobank: Results of a national survey. *PloS One, 11*(7), e0159113; Kraft, S. A., Cho, M. K., Gillespie, K., Halley, M., Varsava, N., Ormond, K. E., … Soo‐Jin Lee, S. (2018). Beyond consent: Building trusting relationships with diverse populations in precision medicine research. *The American Journal of Bioethics, 18*(4), 3–20.

54 Kraft et al., op. cit. note 53.

55 One of the anonymous reviewers makes the excellent point that to support the anticipated clinical outcomes of organoid research, there may be a moral duty for some individuals with very rare diseases and disorders to contribute their samples to a biobank. We do not have scope here to address whether such a moral duty exists and to what extent it applies, but we do wholeheartedly endorse the reviewer's point that biobanks have an interest to encourage such individuals to participate, and that is one reason why biobanks ought to recognize and respond to the individual values, concerns and reasons in a more flexible and relational way than can be facilitated by established consent‐based approaches.

56 O'Neill, O. (2003). Some limits of informed consent. *Journal of Medical Ethics, 29*(1), 4–7.

57 Hofmann et al., op. cit. note 39, p. 15.

58 Lancaster & Knoblich, op. cit. note 1.

59 Boers et al., op. cit. note 7; Bredenoord et al., op. cit. note 3.

60 Hyun, I. (2017). Engineering ethics and self‐organizing models of human development: Opportunities and challenges. *Cell Stem Cell*, *21*(6), 718–720; Boers et al., op. cit. note 43.

61 Boers et al., op. cit. note 7; Bartfeld & Clevers, op. cit. note 3.

62 Boers et al., op. cit. note 43.

63 Boers, S. N., de Winter‐de Groot, K. M., Noordhoek, J., Gulmans, V., van der Ent, C. K., van Delden, J., & Bredenoord, A. L. (2018). Mini‐guts in a dish: Perspectives of adult cystic fibrosis (CF) patients and parents of young CF patients on organoid technology. *Journal of Cystic Fibrosis, 17*(3), 407–415.

64 See, for example, the references in note 2.

65 O'Neill, op. cit. note 56, p. 6.

66 For further details of these challenges, see the references in note 2.

67 Pera, M. F., de Wert, G., Dondorp, W., Lovell‐Badge, R., Mummery, C. L., Munsie, M., & Tam, P. P. (2015). What if stem cells turn into embryos in a dish? *Nature Methods, 12*(10), 917–919; Hyun, op. cit. note 60; Munsie, M., Hyun, I., & Sugarman, J. (2017). Ethical issues in human organoid and gastruloid research. *Development, 144*(6), 942–945; Hyun, I., Munsie, M., Pera, M. F., Rivron, N. C., & Rossant, J. (2020). Toward guidelines for research on human embryo models formed from stem cells. *Stem Cell Reports, 14*(2), 169–174; Piotrowska, M. (2020). Avoiding the potentiality trap: Thinking about the moral status of synthetic embryos. *Monash Bioethics Review, 38*(2), 166–180; Bollinger, J., May, E., Mathews, D., Donowitz, M., & Sugarman, J. (2021). Patients' perspectives on the derivation and use of organoids. *Stem Cell Reports, 16*(8), 1874–1883.

68 Chen, H. I., Wolf, J. A., Blue, R., Song, M. M., Moreno, J. D., Ming, G. L., & Song, H. (2019). Transplantation of human brain organoids: Revisiting the science and ethics of brain chimeras. *Cell Stem Cell, 25*(4), 462–472.

69 Farahany, N. A., Greely, H. T., Hyman, S., Koch, C., Grady, C., Pașca, S. P., … Song, H. (2018). The ethics of experimenting with human brain tissue. *Nature, 556*(7702), 429–432; Lavazza, A., & Massimini, M. (2018). Cerebral organoids: Ethical issues and consciousness assessment. *Journal of Medical Ethics, 44*(9), 606–610; Sawai, T., Sakaguchi, H., Thomas, E., Takahashi, J., & Fujita, M. (2019). The ethics of cerebral organoid research: Being conscious of consciousness. *Stem Cell Reports, 13*(3), 440–447; Hyun, I., Scharf‐Deering, J. C., & Lunshof, J. E. (2020). Ethical issues related to brain organoid research. *Brain Research, 1732*, 146653; Bollinger et al., op. cit. note 67.

70 International Society for Stem Cell Research. (2021). *ISSCR guidelines for stem cell research and clinical translation* (p. 10). Retrieved from https://www.isscr.org/policy/guidelines-for-stem-cell-research-and-clinical-translation

71 Saini, op. cit. note 3; Kraft et al., op. cit. note 53.

72 Burke, W., Beskow, L. M., Trinidad, S. B., Fullerton, S. M., & Brelsford, K. (2018). Informed consent in translational genomics: Insufficient without trustworthy governance. *The Journal of Law, Medicine & Ethics*, *46*(1), 79–86, 81.

73 Ploug,T., & Holm, S. (2017). Clinical genome sequencing and population preferences for information about ‘incidental’ findings—From medically actionable genes (MAGs) to patient actionable genes (PAGs). *PLoS One*, *12*(7), e0179935.

74 Ibid.

75 Given the state of the art, it is difficult not to turn to hypothetical scenarios that speak more to sci‐fi than science when discussing organoid‐based regenerative medicine. So, in what follows, we will confine our discussions to those innovations already in or near development.

76 Chen et al., op. cit. note 68.

77 Bredenoord, A. L., & Braude, P. (2010). Ethics of mitochondrial gene replacement: From bench to bedside. *BMJ*, *341*, c6021; Bredenoord et al., op. cit. note 3.

78 Niemansburg, S. L., Tempels, T. H., Dhert, W. J., van Delden, J. J., & Bredenoord, A. L. (2015). Societal impacts of regenerative medicine: Reflections on the views of orthopedic professionals. *Regenerative Medicine*, *10*(1), 17–24.

79 Boers et al., op. cit. note 63; Bollinger et al., op. cit. note 67.

80 International Society for Stem Cell Research, op. cit. note 70, p. 52.

81 Chen et al., op. cit. note 68; International Society for Stem Cell Research, op. cit. note 70, p. 52.

82 Ibid.

83 Hyun, I. (2015). From naïve pluripotency to chimeras: A new ethical challenge? *Development*, *142*(1), 6–8; Chen et al., op. cit. note 68; International Society for Stem Cell Research, op. cit. note 70, pp. 57–58.

84 Bollinger et al., op. cit. note 67.

85 Kraft et al., op. cit. note 53; Lensink et al., op. cit. note 16. When it comes to ‘downstream control’ over a participant's organoids, Manson observes that this is sometimes equated with a biobank participant having ‘the power to command or order the biobank to do what she wishes’ (Manson, op cit. note 11, p. 542). In this paper, we do not adopt such an approach. Instead, where respect for autonomy is concerned, we argue that biobanks have an autonomy‐based obligation to acknowledge the power a subject has over her decision‐making capacity to determine which kinds of research and clinical applications can make use of her biospecimens. It is in this specific sense that we interpret the notion of ‘downstream control’.

86 Manson, op. cit. note 11, p. 548.

87 Kaye, J., Whitley, E.A., Lund, D., Morrison, M., Teare, H., & Melham, K. (2015). Dynamic consent: A patient interface for twenty‐first century research networks. *European Journal of Human Genetics*, *23*(2), 141–146.

88 Childress, J. F. (2017). Needed: A more rigorous analysis of models of decision making and a richer account of respect for autonomy. *The American Journal of Bioethics*, *17*(11), 52–54.

89 Helgesson, op. cit. note 12, p. 44.

90 O'Neill, op. cit. note 56, p. 6.

91 Ibid.

92 Boers et al., op. cit. note 7; Bredenoord et al., op. cit. note 3.

93 Mostert, M., Bredenoord, A. L., Biesaart, M. C., & van Delden, J. J. (2016). Big data in medical research and EU data protection law: Challenges to the consent or anonymise approach. *European Journal of Human Genetics*, *24*(7), 956–960.

94 Aalto‐Setälä, K., Conklin, B.R., & Lo, B. (2009). Obtaining consent for future research with induced pluripotent cells: Opportunities and challenges. *PLoS Biology*, *7*(2), e42.

95 Christman, op. cit. note 23, p. 158.

96 Benson, P. (1991). Autonomy and oppressive socialization. *Social Theory and Practice, 17*(3), 385–408; Mackenzie, C., & Stoljar, N. (2000). Autonomy refigured. In C. Mackenzie & N. Stoljar (Eds.), *Relational autonomy: Feminist perspectives on autonomy, agency and the social self* (pp. 3–31). Oxford University Press; Anderson & Honneth, op. cit. note 34; Benson, P. (2005). Feminist intuitions and the normative substance of autonomy. In J. S. Taylor (Ed.), *Personal autonomy: New essays on personal autonomy and its role in contemporary moral philosophy* (pp. 124–142). Cambridge University Press; Mackenzie, C. (2008). Relational autonomy, normative authority and perfectionism. *Journal of Social Philosophy, 39*, 512–533.

97 Mackenzie, op. cit. note 96, pp. 518–519.

98 Govier, T. (1993). Self‐trust, autonomy and self‐esteem. *Hypatia*, *8*(1), 99–120; McLeod, C. (2002). Self‐trust and reproductive autonomy. MIT Press; Friedman, M. (2003). *Autonomy, gender, politics*. Oxford University Press; Mackenzie, op. cit. note 96.

99 Christman, op. cit. note 23, p. 143.

100 Christman, J. (1991). Autonomy and personal history. *Canadian Journal of Philosophy, 21*(1), 1–24; Mele, A. (1993). History and personal autonomy. *Canadian Journal of Philosophy, 23*(2), 271–280; Christman, J. (2005). Autonomy, self‐knowledge, and liberal legitimacy. In J. Christman & J. Anderson (Eds.), *Autonomy and the challenges to liberalism: New essays* (pp. 330–358). Cambridge University Press; Christman, J. (2009). *The politics of persons: Individual autonomy and socio‐historical selves*. Cambridge University Press.

101 Meyers, D. T. (2005). Decentralizing autonomy: Five faces of selfhood. In J. Christman & J. Anderson (Eds.), *Autonomy and the challenges to liberalism: New essays* (pp. 27–55). Cambridge University Press, p. 41.

102 Christman (2005), op. cit. note 100, p. 334.

103 Ibid.

104 Ibid.

105 Mele, op. cit. note 100.

106 Friedman, op. cit. note 98; Meyers, op. cit. note 101.

107 Raz, J. (1988). *The morality of freedom*. Clarendon Press, p. 372.

108 For a detailed discussion of how different approaches to information provision promote or undermine the exercise of patient autonomy in clinical situations, see Lewis, J. (2020). Getting obligations right: Autonomy and shared decision making. *Journal of Applied Philosophy*, *37*(1), 118–140, 129–132.

109 Ploug, T., & Holm, S. (2016). Meta consent: A flexible solution to the problem of secondary use of health data. *Bioethics, 30*, 721–732.

110 Raz, op. cit. note 107, p. 375.

111 Kaye et al., op. cit. note 87; Solberg & Steinsbekk, op. cit. note 40; Teare, H. J., Morrison, M., Whitley, E. A., & Kaye, J. (2015). Towards ‘Engagement 2.0’: Insights from a study of dynamic consent with biobank participants. *Digital Health*, *1*, 1–13. https://doi.org/10.1177/2055207615605644; Budin‐Ljøsne, I., Teare, H. J., Kaye, J., Beck, S., Bentzen, H. B., Caenazzo, L., … Mascalzoni, D. (2017). Dynamic consent: A potential solution to some of the challenges of modern biomedical research. *BMC Medical Ethics*, *18*(1), 4.

112 Prictor et al., op cit. note 51.

113 Ibid.

114 Mikkelsen et al., op. cit. note 13.

115 Kaye et al., op. cit. note 87; Solberg & Steinsbekk, op. cit. note 40; Budin‐Ljøsne et al., op. cit. note 111.

116 Prictor et al., op cit. note 51.

117 Solberg & Steinsbekk, op. cit. note 40.

118 Kaye et al., op. cit. note 87.

119 Teare et al., op. cit. note 111.

120 Prictor et al., op cit. note 51.

121 Ibid.

122 Mikkelsen et al., op. cit. note 13.

123 Ibid.

124 Prictor et al., op cit. note 51.

